# Keratokonusdetektion und Ableitung des Ausprägungsgrades aus den Parametern des Corvis®ST: Eine Studie, basierend auf Algorithmen des Maschinenlernens

**DOI:** 10.1007/s00347-020-01231-1

**Published:** 2020-09-24

**Authors:** Achim Langenbucher, Larissa Häfner, Timo Eppig, Berthold Seitz, Nóra Szentmáry, Elias Flockerzi

**Affiliations:** 1 Institut für Experimentelle Ophthalmologie, Universität des Saarlandes, Kirrberger Str., Gebäude 22, 66421 Homburg, Deutschland; 2 Klinik für Augenheilkunde, Universitätsklinikum des Saarlandes, Kirrberger Str., Gebäude 22, 66421 Homburg, Deutschland; 3 Dr. Rolf M. Schwiete Zentrum für Limbusstammzellforschung und kongenitale Aniridie, Universität des Saarlandes, Kirrberger Str., Gebäude 22, 66421 Homburg, Deutschland

**Keywords:** Corvis, Scheimpflug-Hornhauttomographie, Künstliche Intelligenz, Überwachtes Maschinenlernen, Keratokonus, Corvis, Scheimpflug corneal tomography, Artificial intelligence, Supervised machine learning, Keratoconus

## Abstract

**Hintergrund und Zielsetzung:**

In den vergangenen Jahren wurden zunehmend Systeme der künstlichen Intelligenz in der Medizin etabliert, die Pathologien oder Erkrankungen erkennen oder von komplementären Erkrankungen abgrenzen. Bisher liefert das Corvis®ST (Corneal Visualization Scheimpflug Technology, Oculus, Wetzlar, Deutschland) einen Index-CBI, der quasi binär Keratokonus klassifiziert, aber kein Staging zulässt. Ziel der Studie ist es, anhand von Messgrößen des Corvis®ST ein Vorhersagemodell zu entwerfen, das den Topographic Keratoconus Classification Index (TKC) der Pentacam high resolution (HR, Oculus) nachbildet.

**Patienten und Methoden:**

Es wurden 60 Messungen an Normalprobanden (TKC 0) und 379 Augen mit Keratokonus (TKC 1 bis 4) in die Studie mit einbezogen. Nach der Messung mit der Pentacam HR (Zielgröße TKC) wurde eine Untersuchung mit dem Corvis®ST durchgeführt, aus der 6 Messparameter extrahiert wurden, die in den Corvis Biomechanical Index CBI eingehen (ARTh, SP-A1, DA-Ratio 1 mm, DA-Ratio 2 mm, A1 velocity, max. Deformation Amplitude). Neben dem TKC als Zielgröße wurde der binarisierte TKC (1: TKC 1 bis 4, 0: TKC 0) modelliert. Als Gütemaß wurde die Genauigkeit des Modells als Anteil der korrekten Klassifizierungen herangezogen. Fehlklassifizierungen wurden in der Modellierung so bestraft, dass die Abweichung des modellierten TKC-Wertes vom gemessenen Wert bewertet wurde.

**Ergebnisse:**

Es wurden 24 verschiedene Modelle des überwachten maschinellen Lernens aus 6 Familien getestet. Für die Modellierung des TKC in Stufen von 0–4 zeigte das Modell, basierend auf einer Support Vector Machine (SVM) mit linearem Kernel, die beste Performance mit einem Anteil an richtigen Klassifizierungen von 65,1 %. Für den binarisierten Wert des TKC zeigte ein Decision Tree mit grober Auflösung die beste Performance mit einem Anteil an richtigen Klassifizierungen von 95,2 %, direkt gefolgt von der SVM mit linearem oder quadratischem Kernel und dem Nearest Neighborhood Classifier mit kubischem Kernel (jeweils 94,5 %).

**Schlussfolgerungen:**

In der Arbeit soll das Prinzip des überwachten Maschinenlernens in der Anwendung auf die modellierte Klassifizierung von Messbefunden gezeigt werden. So wurden Messdaten des Corvis®ST dazu verwendet, die Einteilung in den Schweregrad eines Keratokonus mittels Pentacam (TKC) mit einer ganzen Reihe von Algorithmen des maschinellen Lernens nachzubilden.

## Abstract (en)

**Background and objective:**

In the last decades increasingly more systems of artificial intelligence have been established in medicine, which identify diseases or pathologies or discriminate them from complimentary diseases. Up to now the Corvis®ST (Corneal Visualization Scheimpflug Technology, Corvis®ST, Oculus, Wetzlar, Germany) yielded a binary index for classifying keratoconus but did not enable staging. The purpose of this study was to develop a prediction model, which mimics the topographic keratoconus classification index (TKC) of the Pentacam high resolution (HR, Oculus) with measurement parameters extracted from the Corvis®ST.

**Patients and methods:**

In this study 60 measurements from normal subjects (TKC 0) and 379 eyes with keratoconus (TKC 1–4) were recruited. After measurement with the Pentacam HR (target parameter TKC) a measurement with the Corvis®ST device was performed. From this device 6 dynamic response parameters were extracted, which were included in the Corvis biomechanical index (CBI) provided by the Corvis®ST (ARTh, SP-A1, DA ratio 1 mm, DA ratio 2 mm, A1 velocity, max. deformation amplitude). In addition to the TKC as the target, the binarized TKC (1: TKC 1–4, 0: TKC 0) was modelled. The performance of the model was validated with accuracy as an indicator for correct classification made by the algorithm. Misclassifications in the modelling were penalized by the number of stages of deviation between the modelled and measured TKC values.

**Results:**

A total of 24 different models of supervised machine learning from 6 different families were tested. For modelling of the TKC stages 0–4, the algorithm based on a support vector machine (SVM) with linear kernel showed the best performance with an accuracy of 65.1% correct classifications. For modelling of binarized TKC, a decision tree with a coarse resolution showed a superior performance with an accuracy of 95.2% correct classifications followed by the SVM with linear or quadratic kernel and a nearest neighborhood classifier with cubic kernel (94.5% each).

**Conclusion:**

This study aimed to show the principle of supervised machine learning applied to a set-up for the modelled classification of keratoconus staging. Preprocessed measurement data extracted from the Corvis®ST device were used to mimic the TKC provided by the Pentacam device with a series of different algorithms of machine learning.

Künstliche Intelligenz hat in den vergangenen Jahren in viele Disziplinen der Medizin, aber auch z. B. in Bereiche der Ingenieurswissenschaften, der Naturwissenschaften oder im Finanzbereich Einzug gehalten [1, 3, 9, 28, 34]. Immer wenn logische Zusammenhänge zwischen Einflussfaktoren und Wirkung nicht mehr klar erkennbar sind oder die Zusammenhänge zu komplex erscheinen, wird heute versucht, mit Verfahren der künstlichen Intelligenz Verbindungen herzustellen. Allen Ansätzen gemein ist, dass die Algorithmen einem Lernprozess unterzogen sind und, auf einer großen Zahl von Datensätzen basierend, Zusammenhänge zwischen Eingangsgrößen herstellen.

Maschinelles Lernen ist ein Unterbegriff der künstlichen Intelligenz und gliedert sich in 2 Bereiche: Beim überwachten Lernen wird Expertenwissen in Form einer manuellen Klassifikation (das sog. „Labeling“ von Datensätzen) hinterlegt. Derartige Algorithmen dienen z. B. dazu, Erkrankungen zu erkennen oder den Schweregrad einer Pathologie zu klassifizieren (klassifizierende Systeme des überwachten Lernens) [3, 11, 28, 36]. Wichtigste Vertreter sind hier Ansätze wie Decision Trees, Support Vector Machines (SVM), Discriminance Analysis, Naive Bayes oder Nearest Neighbourhood [1, 3, 9].

Alternativ kann die Zielgröße des Algorithmus anstatt einer kategorialen Variablen auch eine metrische Variable sein (z. B. ein Keratokonusindex)[16, 19–21, 26, 27, 32, 37], man spricht dann von einem Regressionsalgorithmus des überwachten Lernens. Wichtigste Vertreter sind hier die Linear Regression (Generalized Linear Models [GLM]), Support Vector Regression (SVR), Ensemble Methods, Regression Trees oder Neural Networks. Neben den überwachten Ansätzen des Maschinenlernens gibt es die Option des nicht überwachten Lernens, bei dem kein Expertenwissen in Form eines „Labeling“ von Datensätzen eingebracht wird, sondern der Algorithmus selbstständig versucht, Muster und Strukturen zu erkennen und Datensätze zu gruppieren (sog. Clustern). Die wichtigsten Ansätze in diesem Zusammenhang sind kMeans oder kMedoids, Hierarchical, Gaussian Mixture, Neural Networks oder Hidden Markov Models.

Beim überwachten Lernen ist neben der großen Anzahl an Datensätzen für den Lernprozess von elementarer Bedeutung, dass die Klassifizierung durch einen Experten (das „Labeling“) sehr sorgfältig durchgeführt wird. Sind Datensätze fehlerhaft klassifiziert, so wird der Algorithmus Zusammenhänge fehlerhaft ableiten, was am Ende zu falschen Ergebnissen führt. Weiter ist darauf zu achten, dass bei Klassifizierungsverfahren für den Lernprozess von allen Klassen genügend Datensätze zur Verfügung stehen. Die Datensätze werden zunächst z. B. durch Ziehen einer Stichprobe in einen Lerndatensatz und einen Validierungsdatensatz aufgeteilt. Der Lerndatensatz dient dazu, den Algorithmus zu trainieren, wohingegen der Validierungsdatensatz dazu dient, die Güte des Verfahrens im Sinne von (richtig positiven und richtig negativen) Entscheidungen zu prüfen. Nimmt man diese Aufteilung nicht vor und validiert mit dem Datensatz, mit dem das System vorab trainiert wurde, wird die Performance des Algorithmus in der Regel überschätzt. In den vergangenen Jahren haben sich neben dem einfachen „Holdout“ für die Kreuzvalidierung [9] zunehmend Verfahren wie die „K-Fold“-Kreuzvalidierung oder das „Repeated Random Subsampling“ etabliert. Bei der „K-Fold“-Kreuzvalidierung werden die Daten in K Partitionen aufgeteilt und in K Durchläufen jeweils an K‑1 Partitionen trainiert und am beim Training ausgesparten Datensatz validiert. Beim „Repeated Random Subsampling“ wird in mehreren Durchläufen jeweils eine zufällige Stichprobe als Validierungsdatensatz abgetrennt und am verbleibenden Datensatz trainiert, hier besteht allerdings die Gefahr, dass einzelne Daten bei der Stichprobenziehung in keinem der Durchläufe für das Training oder die Validierung herangezogen werden. Bei der „K-Fold“-Kreuzvalidierung wird am Ende jeder Datensatz K‑1-mal für das Training und 1‑mal für die Validierung herangezogen, trotz höheren Rechenaufwands reduzieren sich hier die Anforderungen an den Umfang des Datensatzes.

Geräteassistiertes Keratokonusscreening basiert heute in der Regel auf Messdaten der Hornhauttopographie, der Hornhauttomographie, der Aberrometrie oder sog. biomechanischen Kenngrößen. Die Hornhauttopographie mit Placidosystemen kann bereits auf eine 30-jährige Tradition zurückblicken, und in den 1990er-Jahren wurden bereits sehr einfach strukturierte Verfahren des Maschinenlernens wie neuronale Netze oder Entscheidungsbäume/Expertensysteme entwickelt (z. B. KPI, KCI, KISA, I‑S bei den TMS-Systemen der Firma Tomey), die dem Anwender eine Empfehlung an die Hand geben, ob bei der individuell vorliegenden Messung von einem Keratokonusbefund (oder Keratokonusverdacht) auszugehen ist [4, 13, 37], mit welcher Wahrscheinlichkeit ein Keratokonus vorliegt [12] oder mit welchem Schweregrad die Pathologie vorliegt [6, 7, 19]. Im einfachsten Fall wurden direkt Messgrößen der Topografie abgefragt und über Grenzwerte eine Entscheidung getroffen (z. B. zentraler Brechwert CK >47,2 dpt → Keratokonus).

Für das Corvis®ST wird mit dem Corvis Biomechanical Index CBI eine Kenngröße angeboten [2, 33], die aufgrund der Architektur des Algorithmus eine quasi binäre Entscheidung trifft, ob ein Keratokonus vorliegt oder nicht [8, 15, 26, 30]. Ein Staging, das beispielsweise bei einer Verlaufskontrolle unabdingbar ist, leistet der CBI nicht. Der TKC-Wert der Pentacam beschränkt sich zwar auf die Analyse der Hornhautvorderfläche, er ist jedoch als Staging-Parameter sehr beliebt, da der Wert sehr stark an die klinische Klassifizierung nach dem Amsler-Krumeich-Schema angelehnt ist, das seit vielen Jahren in der Keratokonusdiagnostik etabliert ist und bei vielen Verlaufsstudien zugrunde gelegt wurde.

In der vorliegenden Arbeit soll anhand von klinischen Daten, die mit dem Corvis®ST-System sowie der Pentacam HR erhoben wurden, skizziert werden, wie aus den DCR-Parametern [2, 33] des Corvis®ST, basierend auf Strategien des überwachten Lernens, ein klassifizierender Algorithmus definiert werden kann, mit dem eine binäre Entscheidung für das Vorliegen eines Keratokonus oder eine Klassifikation des Keratokonus möglich ist. Das „Labeling“ der Datensätze durch einen Experten soll dabei durch das Ergebnis des „Topographic Keratoconus Classification Index“ (TKC) der Pentacam ersetzt werden.

## Patienten und Methoden

Bei 60 Augen von 60 Normalprobanden (30 linke und 30 rechte, mittleres Alter 31,9 ± 12,0 Jahre) ohne Anzeichen eines Keratokonus sowie 379 Augen von Patienten mit Keratokonus (179 linke und 200 rechte Augen, mittleres Alter 35,2 ± 12,5 Jahre) wurde mit der Pentacam HR (Fa. Oculus, Wetzlar, Deutschland) die Hornhauttomographie gemessen. Direkt im Anschluss wurde mit dem im Funktionenumfang erweiterten Luftdrucktonometer Corvis®ST (Fa. Oculus, Wetzlar, Deutschland) gemessen [10, 26, 29]. Die 60 Normalprobanden rekrutierten sich aus der Belegschaft der Klinik für Augenheilkunde am Universitätsklinikum des Saarlandes, die 379 Augen mit Keratokonus wurden aus dem Homburger Keratokonus Center (HKC) ausgewählt. Bei den Normalprobanden war der TKC-Wert „–“ (hier als 0 gewertet). Unter den Patienten mit Keratokonus waren 100 Befunde mit einem TKC-Wert von 1; 144 mit einem TKC-Wert von 2; 117 mit einem TKC-Wert von 3 und 18 mit einem TKC-Wert von 4 gemäß der Klassifikation der Pentacam auf der Basis der Hornhautvorderflächenbefunde. Für die binäre Klassifikation wurden die Befunde mit TKC = 0 zu einem binären TKCbin = 0 und die Befunde mit TKC = 1, 2, 3 oder 4 zu TKCbin = 1 zusammengefasst.

Das Corvis®ST ist ein Luftdrucktonometer, das einen normierten Luftstoß auf die Hornhaut appliziert. Mit einer Hochgeschwindigkeitskamera, die zusammen mit der horizontalen Spaltbeleuchtung (Wellenlänge 455 nm) die Konditionen an eine Scheimpflug-Abbildung erfüllt, wird in kurzer Folge eine Bildsequenz mit 4330 Bildern erfasst und so das dynamische Verhalten der durch den Luftstoß induzierten Eindellung sowie Relaxation der Hornhaut erfasst. Dabei wird für die Messung eine Vielzahl von DCR-Parametern (Dynamic Response Parameter) erfasst. Aus diesen DCR-Parametern werden 6 Größen für die Berechnung des Corvis®ST Biomechanical Index CBI verwendet [33] bzw. fließen in Kombination mit einer Messung der Pentacam in die Berechnung des Topographic Biomechanical Index TBI ein:

- ARTh: [2]: Ambrósio Relational Thickness horizontal, relative Hornhautdickenzunahme vom Zentrum zur Peripherie,
- SP-A1 [2]: Messgröße für die Steifigkeit der Hornhaut, Verhältnis aus Kraft (Druckpuls) zur Verformung der ersten Applanation. SPA1 verringert sich, je höher der Grad des Keratokonus, da die Gesamtsteifigkeit der Hornhaut reduzierter ist,
- DA-Ratio 1 mm [2, 33]: Verhältnis der zentralen Deformation zur peripheren Deformation (1 mm peripher), vergrößert sich mit geringerer Steifigkeit der Hornhaut (mehr „Flattern“ in der Peripherie),
- DA-Ratio 2 mm [2, 33]: Verhältnis der zentralen Deformation zur peripheren Deformation (2 mm peripher), vergrößert sich mit geringerer Steifigkeit der Hornhaut (mehr „Flattern“ in der Peripherie),
- A1 velocity (m/s): Geschwindigkeit der Hornhaut zur ersten Applanation,
- max. Deformation Amplitude (mm): maximale Deformation der Hornhaut in anterior-posteriore Richtung.

Die Kombination der Messdaten beider Messgeräte (Corvis®ST und Pentacam HR) wurde über die gemeinsame Software-Plattform in eine .csv-Datei überführt und für eine weitere Auswertung umformatiert.

Die Weiterverarbeitung der 439 Datensätze erfolgte in der Interpretersprache MATLAB (MathWorks, Natick, USA, Version 2019b). Der Datensatz wurde über eine Zufallsstichprobe in 5 etwa gleich große Gruppen aufgeteilt (K-Fold-Kreuzvalidierung). Dann wurde für 5 Durchläufe jeweils 1 Gruppe als Validierungsdatensatz interpretiert und der Algorithmus auf der Basis der 4 anderen Gruppen trainiert, bis jede der 5 Gruppen einmal als Validierungsdatensatz verwendet wurde.

Die Datensätze wurden mit folgenden Strategien (Familien an Algorithmen) des Maschinenlernens untersucht:

- Decision Trees
  Entscheidungsbäume dienen der automatischen Klassifikation auf der Basis einer geordneten gerichteten (hierarchische Struktur) Sequenz an Entscheidungen [9]. Die Baumstruktur beginnt stets mit einem Wurzelknoten (erste Entscheidung beim Durchlaufen) sowie einer beliebigen Anzahl innerer Knoten, bei denen jeweils wieder eine Entscheidung nötig ist. In den meisten Fällen sind die Entscheidungen an jeder Verzweigung binär (erlauben nur 2 Optionen). Diese Familie von Algorithmen lässt sich sehr anschaulich grafisch darstellen bzw. in einer Software implementieren. Man unterscheidet die unterschiedlichen Auflösungsstufen dieser Familie an Algorithmen nach der maximalen Anzahl von Verzweigungen (Entscheidungen).
  In dieser Arbeit wurden 3 verschiedenen Auflösungsstufen vorgesehen: grobe/mittlere/feine Auflösung entspricht einer Beschränkung auf 20/40/100 Entscheidungen.
- Discriminance Analysis
  Die Diskriminanzanalyse dient der automatischen Einteilung bzw. Klassifikation in 2 oder mehrere Gruppen auf der Basis von Merkmalen [10, 20]. Die hier verwendeten 6 Merkmale (Parameter des Corvis) treten bei jedem Probanden oder Patienten in einer spezifischen Ausprägung auf. Die Strategie der Diskriminanzanalyse ist es nun, in einem 6‑dimensionalen Merkmalsraum Ebenen zu definieren, durch die die gelabelten Fälle sich optimal trennen lassen im Sinne einer quadratischen Distanz des Merkmalsvektors zur Trennfläche. Bei der linearen Diskriminanzanalyse wird davon ausgegangen, dass die zu unterscheidenden Gruppen gleiche Kovarianzmatrizen aufweisen. Bei der nichtlinearen (z. B. quadratischen) Diskriminanzanalyse wird mit einer nichtlinearen (z. B. quadratischen) Diskriminanzfunktion gearbeitet, die Kovarianzmatrizen der verschiedenen Gruppen sind daher nicht mehr gleich.
  In dieser Arbeit wurden die lineare und die quadratische Diskriminanzanalyse verwendet.
- Naive Bayes Classifiers
  Ein Bayes-Klassifikator (benannt nach dem Mathematiker Thomas Bayes) ordnet einer Corvis-Messung eines Patienten/Probanden einer Klasse zu in der Art, dass die „Kosten“, (also das Strafmaß) am geringsten sind. In unserem Fall wird wiederum ein 6‑dimensionaler Raum aufgespannt, und jede Koordinate in diesem Raum wird einer Klasse zugeordnet [20]. Der Rechenaufwand für den Bayes-Algorithmus ist vergleichsweise hoch, so verwendet man of eine vereinfachte Form (Naive Bayes). Die Vereinfachung wird durch die Entflechtung der „Merkmale und Attribute“ geschaffen, in vielen Fällen führt dieser vereinfachte Ansatz bereits zu einer guten Trennung zwischen den Klassen. Unterschieden wird hier in der Annahme der Dichteverteilungen der einzelnen Merkmale.
  In der vorliegenden Arbeit wurde der Naive-Bayes-Algorithmus mit 2 unterschiedlichen Dichteverteilungen implementiert, einmal auf der Annahme einer Normalverteilung (Gaussian Naive-Bayes) und einmal auf der Kernel Naive-Bayes auf der Basis einer Kernel-Gewichtungsfunktion (als Vertreter einer flexiblen nichtparametrischen Wahrscheinlichkeitsdichteverteilung).
- SVM
  Eine Support Vector Machine kann sowohl für Regressionsnetzwerke wie auch für klassifizierende Netzwerke eingesetzt werden [3]. Im vorliegenden Fall wird wiederum ein 6‑dimensionaler Merkmalsraum aufgespannt. Der Ansatz einer SVM ist es, eine Hyperebene (als Trennebene) zu definieren, umgeben von einem möglichst breiten Korridor, sodass der Fläche benachbarten Fällen zuverlässig Klassen zugeordnet werden können. Der Abstand der einzelnen Fälle entlang des Normalenvektors zur Hyperebene muss demnach möglichst groß sein. Die Charakteristik ist nun, dass ausschließlich die Fälle (Corbis-Messungen) für die Definition der Hyperebene herangezogen werden, die in der unmittelbaren Nachbarschaft der Trennebene liegen (sog. Stützstellen oder „support vectors“), alle weiter von der Trennebene entfernten Fälle werden dabei ignoriert. SVM bieten eine besonders elegante und effiziente Form der Berücksichtigung unterschiedlicher Kernelfunktionen, die als verallgemeinertes Skalarprodukt von Vektoren angesehen werden können. Etabliert sind beim maschinellen Lernen lineare oder polynomiale Kernel oder Gauß-Kernel, die auch als radiale Basisfunktionen (RBF) zu verstehen sind.
  In der vorliegenden Arbeit wurden für die Support Vector Machine ein vereinfachter linearer Kernel, ein polynomialer Kernel in Form eines quadratischen und kubischen Ansatzes sowie ein Gauß-Kernel mit 3 verschiedenen Auflösungsstufen implementiert. Die Auflösungsstufe bezieht sich auf die Skalierung des Kernels.
- Nearest Neighborhood Classifiers
  Dieser Nächste-Nachbarn-Klassifikator [1] basiert auf einem nichtparametrischen Verfahren zur Schätzung der Wahrscheinlichkeitsdichteverteilungen. Geht man im vorliegenden Fall wieder von einem 6‑dimensionalen Merkmalsraum aus, so erfolgt die Klassifikation durch eine Mehrheitsentscheidung der bereits klassifizierten benachbarten Messungen im Merkmalsraum. Zu jedem Fall werden k nächste Nachbarn bewertet, wobei k die Auflösungsstufe beschreibt und in der Regel, um ein Unentschieden in der Mehrheitsfindung auszuschließen, eine ungerade Zahl ist. Wird k zu hoch gewählt, so besteht die Gefahr, dass Messungen in die Klassifikation mit einbezogen werden, die im Merkmalsraum weit weg liegen und einer anderen Klasse angehören, wird k zu klein gewählt, so besteht die Gefahr, dass Rauschen in den Trainingsdaten die Entscheidung beeinflusst. Die Algorithmen sind in der Regel sehr effizient zu implementieren.
  In der vorliegenden Arbeit wurde für den Nearest-Neighborhood-Klassifikator mit einer euklidischen Norm als Abstandsmaß mit k = 3/5/11 (grobe/mittlere/feine Auflösungsstufe) gearbeitet, mit einer Cosinus-Distanz (anguläre Distanz), mit einer kubischen Metrik sowie einer Metrik mit gewichteten Abständen. Die Details zu den verschiedenen Metriken sind in [1] aufgeführt.
- Ensemble Learning
  Ensembleverfahren werden beim maschinellen Lernen eingesetzt, um mit einer endlichen Menge an unterschiedlichen Lernalgorithmen anstatt eines einzelnen Algorithmus zu arbeiten [11, 20]. Bagging kombiniert mehrere komplementäre Vorhersagen von Klassifikationsmodellen, wobei die Ergebnisse der einzelnen Modelle gleich gewertet werden. Beim Boosting werden schwache Klassifikatoren verwendet, um in der Zusammenarbeit der Verfahren einen starken Klassifikator zu erhalten. Sehr oft werden hier als Familien von Klassifikatoren Decision Trees (Boosted Tree, Bagged Tree, Random Undersampling Boosted Tree), Diskriminanzanalyseverfahren (Discriminance Analysis), Nearest-Neighborhood-Verfahren in einem Ensemble zusammengefasst.
  In der vorliegenden Arbeit wurden für die Ensemble-Ansätze ein Boosted Tree bzw. ein Random Undersampling Boosted Tree, ein Bagged Tree, eine Subspace Discriminance Analysis sowie ein Subspace-k-Nearest-Neighborhood-Klassifikator implementiert.

Das Strafmaß für eine Fehlklassifizierung wurde so gewählt, dass auf der Hauptdiagonalen keine Strafpunkte und auf der 1., 2. und 3. Nebendiagonalen jeweils mit 1, 2 oder 3 Strafpunkten gewichtet wurde, sodass eine Abweichung des geschätzten vom beobachteten TKC-Wert linear mit dem Maß der Abweichung bestraft wird. Die Tab. 1 zeigt die Strafpunkte, die für eine Fehlklassifikation des Algorithmus vergeben wurden.

**Table 1:** 

		Mit dem Maschinenlernalgorithmus vorhergesagter Wert des TKC				
		TKC 0	TKC 1	TKC 2	TKC 3	TKC 4
Mit der Pentacam gemessener TKC	TKC 0	*o*	1	2	3	4
	TKC 1	1	*o*	1	2	3
	TKC 2	2	1	*o*	1	2
	TKC 3	3	2	1	*o*	1
	TKC 4	4	3	2	1	*0*

Für die Bewertung der einzelnen Ansätze bei der Abbildung des TKC-Index bzw. des binären TKCbin-Index wurde die Konfusionsmatrix berechnet. Als Gütekriterien für die Bewertung der einzelnen Ansätze wurde aus der Konfusionsmatrix die Genauigkeit (Accuracy) als Anteil der im Validierungsdatensatz richtig entschiedenen Klassifizierungen ermittelt.

Zur Einordnung unserer Ergebnisse wurden zudem der CBI des Corvis sowie der Gesamtindex BAD‑D des Belin-Ambrósio-Keratokonusmoduls (Pentacam) mit erfasst. Für die Darstellung der Konfusionsmatrix für den TKCbin und den CBI/BAD‑D wurde beim BAD‑D ein Trennwert von 1,6 und beim CBI ein Trennwert von 0,5 [33] herangezogen.

## Ergebnisse

Der Altersvergleich zwischen Probanden (TKC = 0) und Patienten mit Keratokonus (TKC = 1 bis TKC = 4) wies keinen signifikanten Unterschied auf (*p* = 0,027). In Tab. 2 sind die Ergebnisse der deskriptiven Auswertung (Mittelwert ± Standardabweichung) der 6 DCR-Parameter des Corvis®ST dargestellt, die hier als Einflussgrößen für die Ableitung eines Schätzmodells für den TKC dienen sollen. Die Auswertung erfolgte separat für die Gruppe der Normalprobanden (mit TKC = 0), die Gruppe mit Keratokonus (TKC = 1 … 4) sowie für das Gesamtkollektiv. Im Vergleich der Gruppe der Normalprobanden mit der Gruppe der Augen mit Keratokonus weisen alle 6 ausgewerteten Parameter einen signifikanten Unterschied auf (jeweils *p* < 0,001, Mann-Whitney-U-Test).

**Table 2:** 

Anzahl *n* = 439	ARTh	SP-A1	DA-Ratio 1 mm	DA-Ratio 2 mm	A1 velocity	Max. Deformation Amplitude
TKC 0	536 ± 162	116 ± 18	1,52 ± 0,06	3,89 ± 0,43	0,13 ± 0,02	1,05 ± 0,11
TKC 1–4	242 ± 128	66 ± 22	1,69 ± 0,10	5,56 ± 1,52	0,17 ± 0,03	1,20 ± 0,16
Gesamt	282 ± 167	73 ± 27	1,66 ± 0,11	5,33 ± 1,53	0,16 ± 0,03	0,18 ± 0,16
*p*-Wert	<0,001	<0,001	<0,001	<0,001	<0,001	<0,001

Wie aus Tab. 3 hervorgeht, sind die 6 Parameter ARTh, SP-A1, DA-Ratio 1 mm, DA-Ratio 2 mm, A1 velocity und max. Deformation Amplitude untereinander stark korreliert (Spearman’s |ρ| ≥ 0,576). Das bedeutet für unseren Ansatz, dass die Größen voneinander abhängen und damit der Informationsgehalt dieser Größen begrenzt ist.

**Table 3:** 

	ρ					
τ	ARTh	SP-A1	DA-Ratio 1 mm	DA-Ratio 2 mm	A1 velocity	Max. Deformation Amplitude
ARTh	1	0,765	−0,785	−0,773	−0,583	−0,502
SP-A1	0,579	1	−0,792	−0,841	−0,761	−0,772
DA-Ratio 1 mm	−0,598	−0,605	1	0,933	0,675	0,678
DA-Ratio 2 mm	−0,590	−0,665	0,789	1	0,786	0,725
A1 velocity	−0,416	−0,570	0,491	0,598	1	0,798
Max. Deformation Amplitude	−0,432	−0,583	0,490	0,540	0,612	1

**Figure 1:**
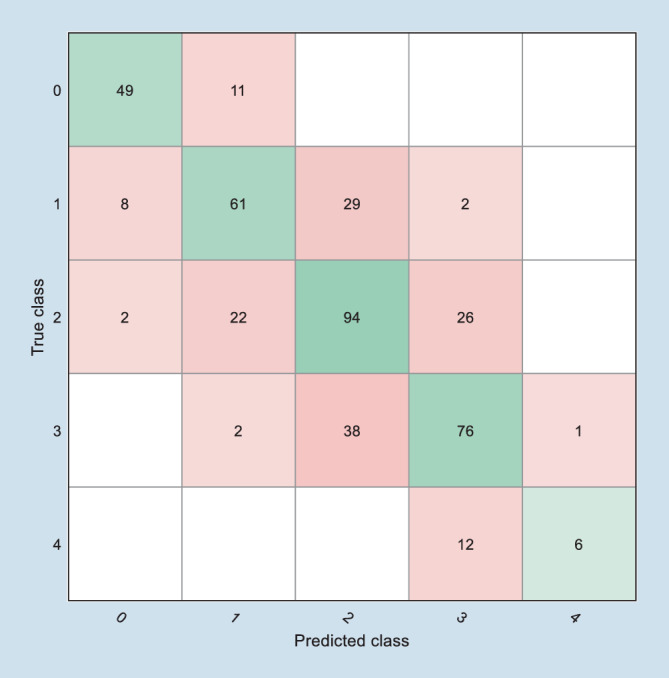

Die Tab. 4 stellt die Ergebnisse der 4 Ansätze des Maschinenlernens mit der besten Performance, angewandt auf die 439 Datensätze zur Nachbildung des TKC, dar. Die 24 Algorithmen aus 6 verschiedenen Familien wurden eingesetzt, um den TKC-Wert der Pentacam nachzubilden, der Werte zwischen 0 (in der Pentacam als „–“ geführt) und 4 annehmen kann. Es zeigt sich, dass bei der Nachbildung des TKC der SVM-Algorithmus mit linearem Kernel die besten Ergebnisse hinsichtlich der Genauigkeit liefert (65,1 % richtige Entscheidungen), gefolgt von der Diskriminanzanalyse mit linearem Kernel und dem SVM-Algorithmus mit Gauß-Kernel in der mittleren Auflösungsstufe sowie der SVM mit quadratischem Kernel. Exemplarisch wurde hier die SVM mit linearem Kernel als der Ansatz mit der besten Genauigkeit dargestellt. Die Abb. 1 zeigt die Konfusionsmatrix mit dem Anteil der richtigen Entscheidungen auf der Hauptdiagonalen. Auf den beiden ersten Nebendiagonalen wird der TKC-Wert der Pentacam jeweils um 1 falsch geschätzt, auf den 2. Nebendiagonalen jeweils um 2 TKC-Stufen daneben etc.

**Table 4:** 

Algorithmusfamilie	Algorithmus	Topographic Keratoconus Classification TKC 0–4 (*n* = 439)	
		Genauigkeit in %	Kumulierte Strafpunkte
Support Vector Machine	Linear	*65,1*	159
	Quadratisch	62,4	178
	Gauß mittel	62,6	174
Discriminance Analysis	Linear	64,0	162

**Figure 2:**
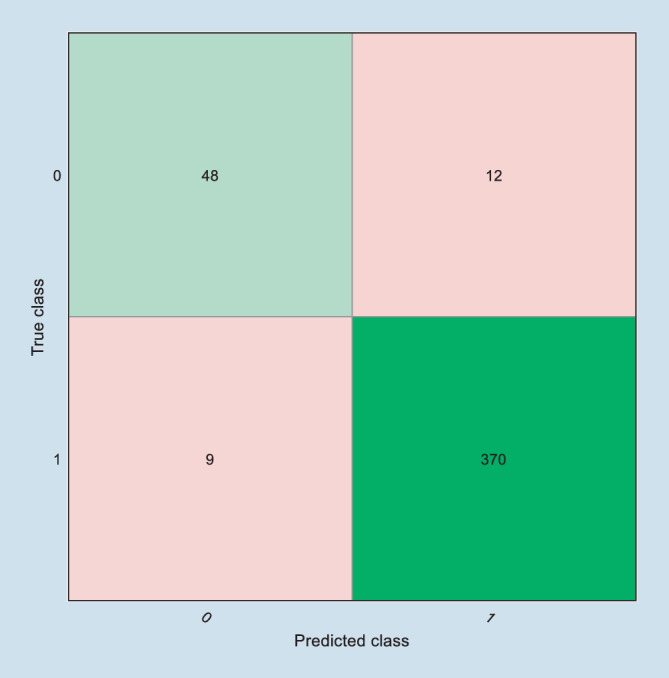

Weiter wurde der binarisierte Wert TKCbin modelliert, der Werte von 0 (in der Pentacam „–“) oder 1 (TKC = 1 bis TKC = 4) annimmt (Tab. 5). Für die Entscheidung, ob ein Keratokonus vorliegt oder nicht (TKCbin), ergab die Modellierung mit dem Entscheidungsbaum in der groben Auflösungsstufe (mit maximal 20 zulässigen binären Entscheidungen) eine Genauigkeit von 95,2 % (Sensitivität 97,4 %, Spezifität 80 %), gefolgt vom SVM-Algorithmus mit linearem oder quadratischem Kernel und dem Nearest Neighbourhood Classifier mit kubischem Kernel (jeweils 94,5 %). Exemplarisch wurde hier aufgrund der besten Performance der Decision Tree mit grober Auflösungsstufe dargestellt. Die Abb. 2 zeigt die Konfusionsmatrix mit dem Anteil der richtig positiven und negativen sowie falsch positiven und negativen Entscheidungen.

**Table 5:** 

Algorithmusfamilie	Algorithmus	Topographic Keratoconus Classification TKC 0–4 (*n* = 439)	
		Genauigkeit in %	Kumulierte Strafpunkte
Decision Tree	Grob	*95,2*	21
Support Vector Machine	Linear	94,5	24
	Quadratisch	94,5	24
Nearest Neighborhood Classifier	Kubisch	94,5	24

**Figure 3:**
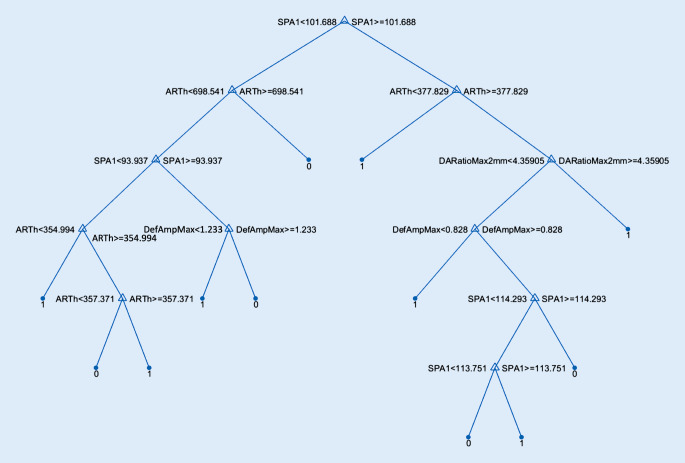

Für den in Abb. 2 herausgegriffenen Ansatz des Decision Tree mit grober Auflösungsstufe wird in Abb. 3 exemplarisch der Entscheidungsbaum grafisch dargestellt. Von den maximal 20 zugelassenen binären Entscheidungen (in der groben Auflösungsstufe) wurden nur 11 benötigt für die Konstruktion des Baumes. An jeder Gabelung wird eine der 6 Einflussgrößen abgefragt, und je nachdem, ob der aktuelle Wert eines Datensatzes größer oder kleiner als der Schwellwert ist, wird der linke oder rechte Ast weiterverfolgt. Nach der letzten binären Entscheidung kann direkt das Ergebnis der Schätzung entweder für 1 (Keratokonus, beschrieben durch einen TKC = 1 bis 4) oder 0 (kein Keratokonus, beschrieben durch TKC = 0 bzw. „–“) abgelesen werden.

Die Tab. 6 zeigt die Konfusionsmatrix des binarisierten TKC (TKCbin) vs. Corvis Biomechanical Index CBI und Gesamtindex des Belin-Ambrósio Moduls der Pentacam BAD‑D. Die Accuracy des CBI ist dabei im Vergleich zu dem hier vorgestellten Index des Maschinenlernens mit 86,8 % deutlich geringer, die des BAD‑D mit 98,9 % deutlich höher. Die entsprechende Konfusionsmatrix des Algorithmus des Maschinenlernens, der auf dem vorliegenden Datensatz die beste Performance erreicht hat, ist in Abb. 2 dargestellt.

**Table 6:** 

	Corvis Biomechanical Index CBI		Belin-Ambrósio Gesamtindex BAD‑D	
TKCbin	0	1	0	1
0	59	1	56	4
1	57	322	4	375

## Diskussion

Neben einer ganzen Reihe an anderen Disziplinen wie der Technik, dem Finanzwesen und den Naturwissenschaften hat die künstliche Intelligenz in den vergangenen Jahren ihren Einzug in der Medizin gehalten [11]. In der Ophthalmologie wird beispielsweise die künstliche Intelligenz verwendet, um Pathologien der Netzhaut zu erkennen und zu klassifizieren, aber auch seit vielen Jahren für die Erkennung und Quantifizierung ektatischer Erkrankungen wie dem Keratokonus, dem Keratoglobus oder der pelluziden marginalen Degeneration. Schon wenige Jahre nach der Einführung der computergestützten Hornhauttopographie als Weiterentwicklung des Videokeratoskops wurden die ersten Screening-Indizes für das TMS‑1 der Firma Computed Anatomy entwickelt, die als Vorfahren dessen angesehen werden können, was heute mit modernen Verfahren möglich ist. In den 90er-Jahren wurden in aller Regel Entscheidungsbäume und einfach neuronale Netze verwendet [12–14, 22–24, 31], heute steht eine umfangreiche Anzahl ganzer Familien von Algorithmen zur Verfügung [20, 21, 25, 27, 32, 35–37].

Entscheidend dafür, welche Verfahren angewandt werden, ist zum einen die Datenbasis selbst, ob z. B. direkt Bilddaten oder Messdaten ausgewertet werden sollen oder ob zum anderen aus Messdaten bereits vorverarbeitete Daten (wie im vorliegenden Fall) herangezogen werden sollen. Werden z. B. Bilddaten als Basis benutzt, so muss zunächst eine deutliche Datenreduktion oder Merkmalsextraktion erfolgen. Weiter muss bei Bilddaten oft erst die translatorische und rotatorische Varianz eliminiert werden und die Größe der relevanten Struktur normiert werden [9]. Aber auch im vorliegenden Fall sieht man anhand der starken Korrelation der Parameter (vgl. Tab. 3), dass bereits bei der geringen Anzahl an Parametern, die für die Modellierung verwendet werden, ein erheblicher Anteil an Redundanz im System vorliegt. Möglicherweise lässt sich das Modell, basierend auf der Korrelation der Einflussfaktoren, weiter vereinfachen (beispielsweise durch eine Hauptachsentransformation und Eliminieren nichtsignifikanter Eigenwerte). Weiter muss geprüft werden, ob bei Ansätzen des Maschinenlernens ein Ansatz aus dem überwachten Lernen zum Einsatz kommen soll oder ein Clustern der Daten beabsichtigt ist, bei dem der Algorithmus selbstständig eine Aufteilung der Daten nach Merkmalen vornimmt. Beim überwachten Lernen wird Expertenwissen benötigt [4–7, 26, 34], also beispielsweise eine Klassifizierung durch einen oder mehrere Experten („Labeling“). Gerade die Einbringung des Expertenwissens macht Verfahren der künstlichen Intelligenz anfällig für Fehlentscheidungen, da Experten im Grenzfall nicht immer kohärente Entscheidungen fällen. Werden mehrere Experten um eine Klassifizierung gebeten, werden klare Fälle meist kohärent bewertet, bei Grenzfällen können Entscheidungen aber abweichen [4–6, 17, 18]. Bei Regressionsverfahren soll am Ende nicht eine Klassifizierung von Datensätzen vorgenommen werden, sondern eine Zahl (z. B. ein Schweregrad einer Erkrankung) stehen. Wichtig ist stets, dass die Daten, mit denen ein Algorithmus trainiert wird, den gesamten Bereich der Möglichkeiten abdeckt, sonst können in Grenzfällen Entscheidungen vom Algorithmus nicht oder nur fehlerhaft getroffen werden. Weiter ist Voraussetzung für eine Bewertung eines Modells, dass die Daten, die für das Trainieren des Modells verwendet werden, disjunkt zu denen sind, die später für die Validierung eingesetzt werden. Wird das nicht beachtet, so wird die Performance des Modells überbewertet.

In der vorliegenden Arbeit soll gezeigt werden, wie man grundsätzlich an die Aufgabenstellung herangeht, aus den vorverarbeiteten Messdaten eines Systems (hier das Corvis®ST der Firma Oculus) ein Modell zu entwickeln, das möglichst gut die Klassifizierung eines anderen Messsystems (hier die Pentacam HR der Firma Oculus) in Form des Topographic Keratoconus Classification Index (TKC) in Stufen von 0 = kein Keratokonus bis 4 = schwerer Keratokonus nachbildet. Ziel ist es, anhand der Parameter des Corvis®ST einen modellierten Parameter abzuleiten, der in möglichst vielen Fällen kohärent zum TKC-Wert ist. Die hier getesteten Modelle werden auf der Basis des (nicht sehr umfangreichen) Datensatzes der Aufgabenstellung nur zum Teil gerecht. Eines der 24 getesteten Verfahren ist in der Lage, gut 65 % richtige Entscheidungen zu treffen. Toleriert man eine Abweichung des modellierten TKC-Wertes von der Ausgabe der Pentacam um 1 Stufe, so werden am Ende nur 6 von 439 Fällen mit einer Abweichung im TKC von mehr als 1 klassifiziert (s. Abb. 1, jeweils 2. Nebendiagonalen). Gibt man sich mit einer Binarisierung im Sinne einer Entscheidung Keratokonus oder kein Keratokonus auf der Basis des TKC zufrieden, so werden über 95 % richtige Entscheidungen getroffen. Hier reicht ein einfacher Entscheidungsbaum mit einer groben Auflösung aus, wie er in Abb. 3 gezeigt ist. Vergleicht man die Kohärenz der Entscheidungen, die man erhält, wenn man mit dem CBI einen Trennwert von 0,5 annimmt [33] oder mit dem BAD‑D einen Trennwert von 1,6, so ergibt sich damit eine Accuracy von 86,8 % für den CBI bzw. 98,9 % für den BAD‑D. Dass Klassifizierungen, die auf denselben Messdaten basieren, grundsätzlich einfacher kohärente Ergebnisse bei einer Klassifizierung erzielen können, liegt auf der Hand (BAD‑D vs. TKC, beide basierend auf den Daten der Pentacam). Entsprechend schneiden der CBI sowie der hier vorgestellte Decision Tree schlechter ab. Vergleicht man den CBI mit dem Decision Tree in der groben Auflösungsstufe, so zeigt der CBI eine höhere Spezifität, jedoch eine geringere Accuracy, also eine geringere Anzahl der mit dem TKCbin übereinstimmenden Entscheidungen (381 vs. 418). Über die Anpassung der Entscheidungsschwelle kann ggf. der CBI hinsichtlich der Accuracy noch weiter optimiert werden. Allerdings kann diese Aussage nur auf dem vorliegenden Datensatz getroffen werden.

Bei der vorliegenden Studie wurde mit einer symmetrischen Matrix für das Strafmaß gearbeitet. Das bedeutet, dass eine Unterschätzung des TKC-Wertes genauso behandelt wird wie eine Überschätzung, in unserem Fall wird jede Stufe der Abweichung mit 1 Punkt geahndet (Tab. 1). So wird am Ende die Konfusionsmatrix in etwa diagonal-symmetrisch (s. Abb. 1). Möchte man dagegen ein Modell entwickeln, das z. B. eine hohe Sensitivität hat (damit möglichst kein pathologischer Befund übersehen wird, was natürlich auf Kosten der Spezifität geht), so kann gezielt mit einer asymmetrischen Matrix für das Strafmaß gearbeitet werden.

Zusammenfassend soll diese Studie das prinzipielle Vorgehen zeigen, wie bei einem Ansatz des überwachten maschinellen Lernens eine Klassifizierung (der Goldstandard für das System) durch ein Modell so nachgebildet wird, dass eine möglichst gute Übereinstimmung der vorhergesagten Klasse mit der als „Expertenwissen“ in den Algorithmus eingespeisten Klassifizierung erreicht wird. Das Ergebnis kann anschließend z. B. mit der Konfusionsmatrix bewertet werden, aus der die Rate der richtig klassifizierten Fälle (Genauigkeit, Accuracy) abgelesen werden kann. Das Vorgehen wurde anhand eines Datensatzes von Normalprobanden und Patienten mit Keratokonus aus dem Homburger Keratokonus Center (HKC) gezeigt, bei denen anhand von vorverarbeiteten Messgrößen des Corvis®ST der TKC-Wert der Pentacam nachgebildet werden sollte.
